# Too-often Feeding of Children

**Published:** 1885-08

**Authors:** A. E. Hoadley

**Affiliations:** Professor of Anatomy, Chicago College of Physicians and Surgeons


### TOO-OFTEN FEEDING AS A CAUSE OF DISEASE IN INFANTS, AND A PROTEST AGAINST ONE COW'S MILK.

*By* A. E. HOADLEY, M. D., *Professor of Anatomy, Chicago College of Physicians and Surgeons.*

(Read before the Illinois State Medical Society, at Springfield, May 20th, 1885.)

In the first years of my practice I was sorely tried by that class of infantile maladies which depend upon improper food, artificial or natural, and improper feeding; which diseases constitute by far the greater number of affections from which infants suffer and die.

I lost confidence in therapeutics, as I could get but negative results; and the various kinds of food and schemes of feeding alike disappointed in the majority of the well-defined cases of indigestion or mal-assimilation, diarrhœa and vomiting. They continued to vomit, purge, pine and die, in spite of all the elaborate and scientific preparations, prepared in accordance with the most approved physiological laws. In looking over the ground, it did seem as though there could be nothing wanting to properly nourish, and that without disturbance, the little infant, who at birth was absolutely perfect and healthy.

Still something was wrong, so I began a systematic inquiry to discover the error, and I very soon found that there was great discrepancy of opinion as to how often a young infant should be fed. Some affirming that four times a day and once in the night was enough, while others advocated feeding often, claiming that a healthy baby would not take more nor oftener than it required. The weight of evidence was in favor of feeding a sick baby, and especially a baby troubled with vomiting, very often and in very small quantities, every one having a favorite combination of food and therapeutics. Here was the error then, and I have by years of patient attention to the subject, proved to my perfect satisfaction that too-often feeding is a greater cause of disease than improper food, and since I have been of that opinion, putting precept into practice, my confidence in therapeutics has been restored. I can now as surely get the desired results from medication when it is indicated, in the infant as in the adult. I said, too-often feeding; I do not by any means wish it to be understood that I am an advocate of long intervals between feeding. On the contrary, I believe the intervals should be as short as possible, allowing sufficient time for the perfect digestion of the previous meal. The time may vary according to the food employed and the quantities taken; but when the shortest time in which a meal can be digested is fixed upon, that should be the minimum interval of time allowed to elapse between feeding, allowing the infant to go a longer time if sleeping or quiet; but under no circumstances should it be allowed to partake of food oftener. Now, what is the common practice? In nine cases out of ten, a baby is nursed or fed every time it cries, even though the crying is induced by an already over-distended stomach.

Some babies save themselves from worse results by promptly vomiting the excess of milk, retaining sufficient to perfectly nourish them, and therefore thrive; while others develop a condition of indigestion amounting to positive dyspepsia, and food induces pain on entering the stomach. Gastro-enteritis, entero-colitis, and a large number of the wasting diseases are induced by this one thing of feeding an infant so often that it cannot digest its food, thus causing mal-assimilation and all of its baneful consequences. The larger number of the diseases thus produced can be cured by simply removing the cause; that is, feed at regular intervals, say once in two hours or longer, but in no case oftener. I have repeatedly seen infants with moderate vomiting and diarrhœa, recover from both promptly and without medicine of any kind from simply regulating the time of feeding without making any material change in the food. I will cite two cases out of many thus treated.

CASE 1st Male baby, age three months. Well nourished and apparently perfect at birth. Mother's milk was found to be insufficient at the close of the second month. Cow's milk was added and occasionally some farinaceous diet, previous to which time the baby was nursing the breast almost incessantly; but as the milk was scanty there was no disturbance. With the addition of the new diet there came a disturbed condition of the bowels, consisting of frequent passages of undigested lumps of curd or caseine, some mucus, streaks of green, sometimes small, at others large and watery. Paregoric was used occasionally in suitable doses to control the diarrhœa. This condition continued with gradually increasing severity, until vomiting was added as a symptom of the deranged digestion.

At this time my attention was called to the case. The child had been vomiting for two days, and now had reached a point where it threw up everything that it took into its stomach, and it would take anything with avidity that was offered to it. There was as yet no fever, but restlessness and a flabby and shrunken condition of the tissues.

The course of the disease thus far had been very gradual, and there was not as yet any lesion, no positive pathological condition, being simply a deranged function. To remove the cause would be to successfully treat the case.

"How often do you feed this baby?" I asked.

"I feed him all the time, you might say, for he is never quiet unless he is eating," was the answer.

I tested the cow's milk with litmus paper and found it sour, although it was just milked from a select cow kept in the city close at hand. Ordered two teaspoonfuls of lime water to be added, to neutralize the acidity of the milk, and that it be fed to the baby every two hours, and, under penalty, no oftener.

As might be expected, the first was vomited, and the little sufferer had no alternative but to thirst and starve and cry in consequence, for two long hours, when he was again fed three ounces of milk with two teaspoonfuls of lime water added. This was taken with relish, and retained for about one hour when a part of it came up. This was the last time the baby vomited, and in three days the passages were normal in all respects, and the baby was "the best natured little darling you ever saw."

Did the lime water do it? I think not, inasmuch as they had been using lime water all the day before, and for several days had been administering pepsin with a view to correct the already recognized condition of indigestion.

The lime water was dicontinued, and its diet, consisting of cow's milk, crackers and oatmeal, with breast milk at night only, was continued under the new regulations, and the child has remained well until the present time, three months, and has resumed its usual plumpness. In this case the mixed diet of sour cow's milk, etc., might be assigned as a good cause for the severe derangement of the stomach and bowels; but upon instituting regularity of feeding, the patient did well without change.

On the other hand, if the diet had been changed and the pernicious practice of feeding all the time continued, it is my conviction, derived from sad experience, that it would have been simply a question of constitutional strength against a constant irritant, assisted perhaps by therapeutics.

CASE 2nd. A fine healthy baby at birth, female, well developed and well nourished. The mother's milk came early and in abundance, and from ordinary examination seemed to be of excellent quality. The baby was inclined to worry some from the first; was supplied with this best of all nourishment in abundance, and nearly all the time, except when asleep, for the purpose of keeping it quiet. She would vomit a part of nearly every meal, and then nurse again. At the end of six weeks she began to have frequent bad passages from the bowels, which were followed by vomiting in good earnest, with straining and gagging and great distress. At this stage I prescribed that the baby be put to the breast once in two hours, and at first be allowed to take but a small quantity, and when vomiting ceased to take as much as she would, instructing the mother to take the baby away from the breast as soon as she began to play with it.

This baby recovered promptly, and in less than a week had forgotten its bad habits. These cases were thus treated for the purpose of demonstrating that too-often feeding was the cause of the attack and not because other remedies would not be serviceable. On the contrary, therapeutical agents give the most gratifying results when combined with the above plan of regular feeding.

In this connection I wish to enter a protest against the popular practice of recommending one cow's milk for babies. This may be judiciously done in the country, but in towns, and especially in the larger cities, there are so many objections to the practice that it is fraught with danger to the little ones.

The selection of the cow is rarely if ever made by the physician; he cannot devote the time necessary to inspect cow-sheds and cows and examine the milk and look for possible causes of irritation and infection; the selection is usually made without the advice of the physician, and when his attention is called to the fact, the following are some of the reasons why he should not assent: the cow may be too old or too young, may have been milked too long or not long enough, since she calved. This time should nearly correspond to the age of the child to be fed. The milk may be extremely rich in butter or casein or both, or very poor in these constituents, as cow's milk varies in its quality to a greater degree than human; or the milk may possess some peculiarity of its own, which will by slow degrees injure the child. Furthermore, the manner in which town-cows are kept and fed exerts a pernicious influence. They are usually fed all the available garbage, which is mostly rotten, and refuse of the kitchen, and still-slop, which is next to garbage in cheapness and only one degree less objectionable. They are, as a rule, kept in low, damp and dark sheds or stables, without exercise, from the time of heavy frosts in the fall until grass starts in the spring. A large proportion of them are not provided with hay or straw for bedding, or dry bedding of any kind. They are compelled to lie down in their own liquid excrement, in which their bags and teats macerate until they become covered with sores. The filth adheres to their bodies, except shoulders and back, forming a hard crust, which only falls off in the spring, taking the hair with it, leaving the skin naked, which is frequently covered with little sores or marks where sores have been. Some of these poor animals can scarcely face the daylight when first turned out to graze, owing to their long confinement in the dark. They are so weak that it is with great difficulty that they can get up alone, and very frequently it becomes necessary to assist them to rise. They will tremble all over after walking a mile when they are first driven out to pasture. Still they will retain a fair degree of flesh, and there are but few deaths, their food being sufficient in amount but bad in quality. Is this condition of things conducive to the production of good, health-giving milk? I think not. On the contrary, I believe that milk from such sources has been the direct cause of irremediable disease. This condition of things is so common in large towns, that one cow's milk should be regarded with suspicion and not countenanced until it can prove a good character. In the summer-time, town-cows are over-worked, going to and from their pasture lands; and to compensate for the meagre supply of grass that prevails, they are fed as before on garbage and still-slop. As a result, they give sour milk the year around. The milk of stall-fed cows is apt to be acid in its reaction, but it is always markedly so from town-cows, unless they are fed exclusively on grass and that within easy distance.

On the other hand, it is only necessary to say that at the country milk dairies, the chief source of city supply, everything is done that will tend to the comfort and well-being of the cows. The dairy-men know very well that all extras are more than paid for by a greater yield of milk. The facilities for rapidly cooling and shipping the milk are perfect, so that towns and cities are supplied with morning's milk, which is produced under the most favorable circumstances, within two or three hours from the time it is milked. This milk is handled in eight gallon cans. Each can-full represents the milk from eight or ten cows; the quality must therefore average good in all respects.

In about twenty tests made with litmus paper by myself, at various times, and of milk from different dealers, it has proved to be neutral in its reactions. Should there be one cow that produced poor milk, its influence could not be very detrimental to a child, if mixed with that of eight others. Again, the chances would be a hundred to one that it would not get the same cow's milk two days in succession. It is therefore impossible for any vice to be long continued through milk delivered from a country milk dairy, except that vice be derived from bad management in the care or transportation. The chances of such contamination are reduced to the minimum as compared with one cow's milk.

Every first-class milk peddler is willing to supply his customers with the unadulterated and unskimmed morning's milk for seven cents a quart. The night's milk, after suffering various degrees of skimming and the blue color removed by a small quantity of burnt sugar, is sold by the grocers for from two to five cents a quart.

